# In Situ Generation of Poly (Vinylene Carbonate) Based Solid Electrolyte with Interfacial Stability for LiCoO_2_ Lithium Batteries

**DOI:** 10.1002/advs.201600377

**Published:** 2016-11-10

**Authors:** Jingchao Chai, Zhihong Liu, Jun Ma, Jia Wang, Xiaochen Liu, Haisheng Liu, Jianjun Zhang, Guanglei Cui, Liquan Chen

**Affiliations:** ^1^ Qingdao Industrial Energy Storage Technology InstituteQingdao Institute of Bioenergy and Bioprocess TechnologyChinese Academy of SciencesQingdao266101China; ^2^ University of Chinese Academy of SciencesBeijing100049China; ^3^ College of Chemistry and Molecular EngineeringQingdao University of Science & Technology266042QingdaoChina; ^4^ Beijing National Laboratory for Condensed Matter PhysicsInstitute of PhysicsChinese Academy of SciencesBeijing100190China

**Keywords:** in situ generation, interfacial stability, lithium batteries, poly (vinylene carbonate), solid electrolyte

## Abstract

Nowadays it is extremely urgent to seek high performance solid polymer electrolyte that possesses both interfacial stability toward lithium/graphitic anodes and high voltage cathodes for high energy density solid state batteries. Inspired by the positive interfacial effect of vinylene carbonate additive on solid electrolyte interface, a novel poly (vinylene carbonate) based solid polymer electrolyte is presented via a facile in situ polymerization process in this paper. It is manifested that poly (vinylene carbonate) based solid polymer electrolyte possess a superior electrochemical stability window up to 4.5 V versus Li/Li^+^ and considerable ionic conductivity of 9.82 × 10^−5^ S cm^−1^ at 50 °C. Moreover, it is demonstrated that high voltage LiCoO_2_/Li batteries using this solid polymer electrolyte display stable charge/discharge profiles, considerable rate capability, excellent cycling performance, and decent safety characteristic. It is believed that poly (vinylene carbonate) based electrolyte can be a very promising solid polymer electrolyte candidate for high energy density lithium batteries.

## 1 Introduction

Lithium ion battery has gained extensive and successful application in portable and consumable electronic devices.^1^ However, the conventional lithium ion battery using nonaqueous liquid electrolytes encounters safety issues, i.e., fire or explosion hazards, when they are closely packed into a large format module, especially for electric vehicles. These issues stimulate intensive interest in high safety and high energy density solid state lithium batteries.^2^, ^3^, ^4^ Although some strategies in the modification of separator could improve the performance of lithium ion batteries to some extent,^5^, ^6^ the existence of liquid electrolyte always is closely related to a hidden safety hazard. The solid electrolyte is a key component for ensuring high safety and high voltage window. Among solid state electrolytes, solid polymer electrolyte takes the advantage of high flexibility, easy processability, and low interfacial resistance when compared with inorganic ceramic electrolyte.^7^ These merits qualify them promising materials for high energy solid state battery.

Till now, three categories of solid polymer electrolytes (SPEs) have been extensively reported, i.e., Polyoxyethylene (PEO)based solid electrolyte, succinonitrile based solid electrolyte, and polyester based solid polymer electrolyte.^3^, ^4^, ^6^, ^8^, ^9^, ^10^ Since Wright and Armand discovered that the PEO based electrolyte possessed considerable ionic conductivity above 10^−7^ S cm^−1^, this solid electrolyte system has attracted extensive interests in academic field as well as industrial community.^11^, ^12^ The branched PEO based solid polymer electrolyte has been commercialized by DAISO (Japan). However, the main chains of PEO readily crystallize at room temperature to hamper the ion migration resulting in a lower ionic conductivity at room temperature.^13^, ^14^ Moreover, it is generally regarded that its electrochemically stability window is lower than 4.0 V versus Li/Li^+^, which could be well suitable for LiFePO_4_, but not for the high voltage cathodes.^8^ The succinonitrile based solid electrolytes possessed ultrahigh ionic conductivity above 10^−3^ S cm^–1^ at ambient temperature due to the presence of unique plastic–crystalline phase between –40 °C and 60 °C.^9^ Lee and co‐workers developed a facile strategy to prepare polymer network‐integrated plastic–crystalline electrolyte for flexible solid state LiCoO_2_/Li_4_Ti_5_O_12_ batteries.^15^ However, it was indicated that succinonitrile possessing two nitrile groups might be electrochemically reduced to generate unstable solid electrolyte interface (SEI) on graphite.^16^ In addition, succinonitrile would react with lithium foil anodes. Those drawbacks severely limited its application for high energy solid stable batteries. Some polyester‐based solid polymer electrolytes, such as poly(propylene carbonate)^4^ and poly(ethylene carbonate),^17^, ^18^ have achieved great success in high performance of lithium batteries. It has been found that poly(ethylene carbonate)‐based solid electrolyte showed an ionic conductivity of the order of 10^−5^ S cm^−1^ at 30 °C.^17^ Zhou et al. also reported that poly(methyl methacrylate‐styrene)‐based polymer electrolytes have a number of potential applications in Li‐O_2_ batteries.^19^ However, most of polyester‐based solid polymer electrolytes were made by ex situ solution casting technique, which used a large amount of solvents and increased the fabrication procedures and cost as well. So, it is extremely urgent to seek high performance solid polymer electrolytes that possess both excellent electrochemical properties and facile preparation technique. In situ polymerization was reported to be such a kind of technique for preparing solid polymer electrolyte in lithium batteries.^20^, ^21^ Kang and co‐workers have prepared some gel polymer electrolyte by in situ polymerization, which could obtain high performance polymer electrolyte.^21^ The monomers or precursors are liquid state, which facilitate to be injected into the batteries. In addition, in situ generated solid electrolytes would have an excellent contact and affinity with both electrodes. This strategy simplifies the preparation process of solid polymer electrolyte and cuts the cost of fabrication.

Some SEI additives are frequently used in state‐of‐the‐art nonaqueous electrolytes to enhance the interfacial compatibility toward lithium/graphitic anodes and high voltage cathodes, consequently endowing better cycling performance.^22^, ^23^ Unsaturated compounds have always been selected as such additives in nonaqueous electrolytes, because unsaturated functional groups (double or triple bonds) provide a site for polymerization under electrochemically reductive (or oxidative) conditions. Typically, vinylene carbonate (VC) is one of promising unsaturated additives.^24^, ^25^ It was reported that the VC could be polymerized into poly(vinyl carbonate) (PVCA) on the surface of the graphitic anode during charge/discharge process, resulting in the main ingredients of SEI layers and improved the compatibility of the interfacial and cycling performance of the batteries.^26^ In addition, the presence of VC in solutions would reduce the impedance of cathodes at ambient temperature.^25^ Inspired by the positive interfacial effect of the as‐generated PVCA on interfacial compatibility, it is believed that PVCA based electrolyte can be a very promising solid polymer electrolyte candidate for high energy density lithium batteries.

Herein, this novel PVCA based solid polymer electrolyte was generated via a facile in situ polymerization process. It was demonstrated that this solid polymer electrolyte endowed high voltage LiCoO_2_/Li batteries stable charge/discharge profiles, decent rate capability, and excellent cycling performance, which was believed to be promising solid polymer electrolyte for high energy lithium batteries.

## 2 Results and Discussion

### 2.1 In Situ Polymerization and Characterization of PVCA

**Figure 1:**
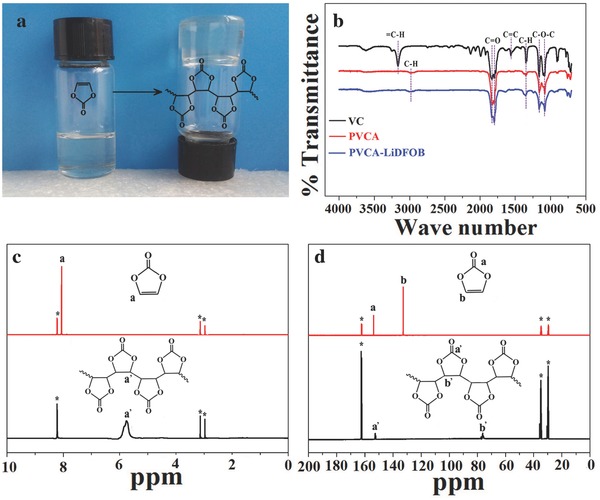
a) The typical image of in situ polymerization of VC into PVCA after heating at 60 °C for 24 h; b) FTIR spectra comparison of VC, PVCA, and PVCA‐LiDFOB; c) ^1^H NMR spectra of VC and PVCA in DMF‐d_6_, and d) ^13^C NMR spectra of VC and PVCA in DMF‐d_6_.

Liquid VC can be polymerized into PVCA catalyzed by a thermally initialized radical initiator at 60 °C for 24 h (shown in **Figure** **1**a). Fourier transform infrared spectroscopy (FTIR), ^1^H NMR, and ^13^C NMR measurements were conducted to analyze the chemical structure of PVCA. As can be seen from the FTIR spectra comparison in Figure 1b that after polymerization the absorption peak at 3166 cm^−1^ disappeared and a new peak occurred at around 2976 cm^−1^, which were well assigned to the chemical structure change of the C=C double bond into C—C single bond. The chemical structure changes of VC to PVCA after in situ polymerization were further confirmed by ^1^H NMR and ^13^C NMR spectrum (seen in Figure 1c,d). After polymerization, the proton chemical shift of CH=CH double bond was shifted from 8.0 to 5.7 ppm, and the carbon chemical shift was shifted from 130 to 76 ppm, respectively. Both FTIR and NMR spectra verified the chemical structure of PVCA.^27^ Gel‐permeation chromatograph indicated that the weight‐average molecular weight (*M*_w_) of PVCA could be up to 4.8 × 10^5^ and the polydispersity (PDI, *M*_w_/*M*_n_) was 2.77 (seen in Figure S1, Supporting Information). Lithium difluoro(oxalate) borate (LiDFOB) is a kind of lithium salt used in lithium batteries, possessing the combined chemical structures of lithium bis(oxalate) borate and lithium tetrafluoroborate (LiBF_4_). The LiDFOB‐based electrolytes possessed superior ionic conductivity and elevated temperature property.^23^, ^28^ So, LiDFOB was adopted as lithium salt in this paper. Differential scanning calorimetry (DSC) curve showed that PVCA‐LiDFOB had a slightly higher glass‐transition temperature (*T*_g_) than PVCA (shown in Figure S2, Supporting Information), which was due to the formation of intermolecular interaction between the lithium ion and oxygen atom in C=O. And there is no obvious melting peak for PVCA‐LiDFOB at the temperature range between –50 and 100 °C, which meant that PVCA‐LiDFOB was an amorphous structure. Thermogravimetric analysis curve displayed that PVCA‐LiDFOB had a superior thermal stability that being negligibly volatile until a high temperature of 250 °C, which was of great significance for the safety improvement of lithium batteries. (seen in Figure S3, Supporting Information). The first weight loss at around 100 °C was related to the evaporation of trace water present in the sample. In addition, there was no crystallization peak in X‐ray diffraction (seen in Figure S4, Supporting Information), which was consistent with the result of DSC.

**Figure 2:**
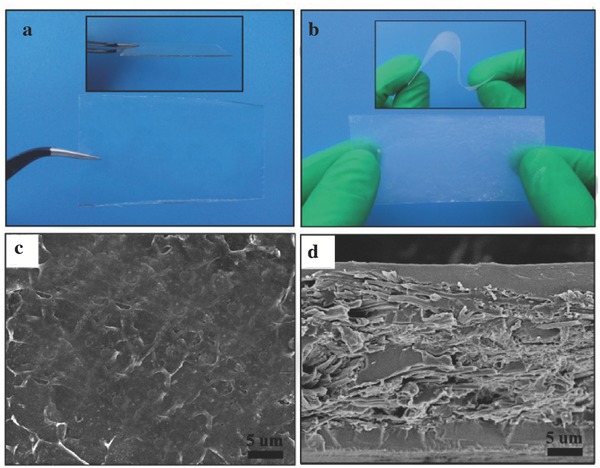
a) The digital images of PVCA‐LiDFOB; b) the digital images of cellulose/PVCA‐LiDFOB composite solid polymer electrolyte; c) the surface morphology and d) the cross‐section of cellulose/PVCA–LiDFOB composite polymer electrolyte.

It could be seen in **Figure** **2**a that PVCA‐LiDFOB was transparent. After incorporating into a cellulose nonwoven substrate, the composite cellulose/PVCA‐LiDFOB solid polymer electrolyte (hereafter abbreviated as “PVCA‐SPE”) became translucent due to cellulose fibers' induction (shown in Figure 2b). Scanning electron microscope (SEM) was used to observe the surface morphology and cross‐section of PVCA‐SPE. It could be seen from Figure 2c that the surface of PVCA‐SPE was quite smooth, which meant that PVCA‐LiDFOB had developed a dense coating layer on the surface of cellulose nonwoven substrate. The dense coating layer would improve solid/solid interface contact between electrolyte and electrode. In addition the cross‐section image showed that the thickness of obtained PVCA‐SPE was about 30 µm, which was very close to that of cellulose‐based Supporting Information. Moreover, the in situ generated PVCA‐LiDFOB solid polymer electrolyte uniformly incorporated into the voids of cellulose nonwoven, resulting in interconnected channels for lithium ions transportation.

**Figure 3:**
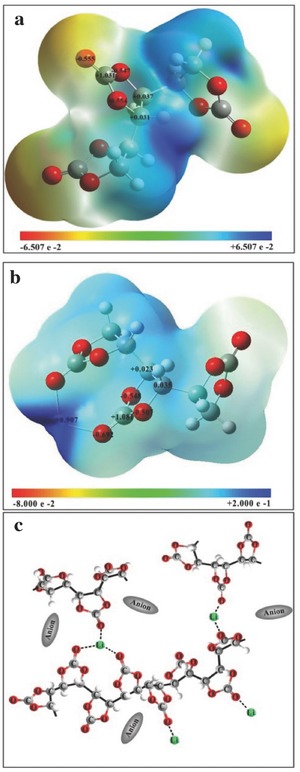
a) Probability of electron cloud density distribution of PVCA (three repeating units of PVCA) and b) Probability of electron cloud density distribution of PVCA with Li^+^; c) possible interaction of Li^+^ with carbonate group in PVCA.

To further investigate the interaction between Li^+^ with carbonate group in PVCA, probability of electron cloud density distribution of the repeating unit of PVCA was calculated by Gaussian software (shown in **Figure** **3**a,b). Natural bond orbital charge of oxygen atom in C=O was –0.555, which was slightly lower than that of the oxygen atom in C—O—C (–0.547 and –0.554), indicating that Li^+^ trended to interact with both of them (seen in Figure S5, Supporting Information). However, steric hindrance effect would probably weaken the interaction of Li^+^ with oxygen atom in C—O—C, conversely favoring of Li^+^—O=C (seen in Figure 3c). The segmental motion of the PVCA along with this Li^+^ coupling/decoupling with oxygen atom in C=O mainly contributed to the lithium ion conductivity. This similar mechanism was also previously discussed by Tominaga et al. and Zhang et al.^27^, ^29^

### 2.2 Electrochemical Properties of PVCA‐SPE

**Figure 4:**
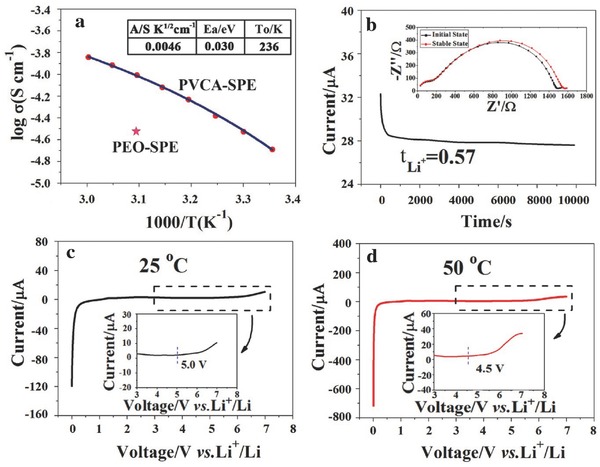
a)Temperature dependent ionic conductivity of PVCA‐SPE; b) current variation with time during polarization of a Li/PVCA‐SPE/Li symmetrical cell at 25 °C, with total applied potential difference of 0.05 V. Inset shows the AC impedance spectra of symmetrical battery. Linear voltammetry curve of Li/PVCA‐SPE/SS at c) 25 °C and d) 50 °C.

Azobisisobutyronitrile (AIBN) is a very common and efficient initiator for free radical polymerization. The charge/discharge curves of liquid electrolyte based LiCoO_2_/Li batteries with and without AIBN (1.0 mg mL^−1^) were displayed in Figure S6 in the Supporting Information. Obviously, such a small amount of AIBN had almost no impact on electrochemical properties of LiCoO_2_/Li batteries. So, AIBN was adopted as the initiator for the generation of PVCA‐SPE. The temperature‐dependent ionic conductivity of PVCA‐SPE with 1.0 mg mL^−1^ AIBN was shown in **Figure** **4**a. The ionic conductivity of PVCA‐SPE is 2.23 × 10^−5^ S cm^−1^ at 25 °C and 9.82 × 10^−5^ S cm^−1^ at 50 °C, which is higher than that of previous reported PEO based electrolyte and PAN based electrolyte.^14^, ^30^ The ionic conductivity of PEO based electrolyte was only about 2.76 × 10^−5^ S cm^−1^ at 50 °C. The temperature‐dependent ionic conductivity behavior of polymer electrolytes can be described by the Vogel–Tamman–Fulcher (VTF) empirical equation

(1) $$\sigma \;=\;A\;T^{-1/2}\;\exp\left(\frac{-E_{a}}{R\left(T-T_{o}\right)}\right)$$

where *A* is conductivity pre‐exponential factor, which is related to the number of carrier ions, *E*_a_ is the activation energy, *T*_0_ is the Vogel scaling temperature at which the free volume disappears or at which configuration free entropy becomes zero, and *R* is the ideal gas constant.^31^, ^32^ The calculated values of VTF fitting parameters for PVCA‐SPE was shown in Figure 4a. Compared with other kinds of polymer electrolytes,^3^ PVCA‐SPE has a lower activation energy (0.030 eV), indicative of the low energy barrier for lithium ion transfer in PVCA‐SPE.

As we know, in dual ions conducting system, anions of salt usually move much faster than lithium cations, which makes the lithium ion transference number lower than 0.5.^33^, ^34^ In lithium ion battery, only lithium ion is effectively involved into electrode reaction and lithium ion conductivity itself is contributed to the effective conductivity. The lithium ion transference number is a critical factor regarding the ionic conductivity and generally a high transference number is desirable for better power output. The transference number can be calculated from the Bruce–Vincent–Evans equation. As seen from Figure 4b, the current value reached a plateau of 27.6 µA from the initial current value of 32.4 µA at a voltage of 0.05 V. It should be noted that there are two semicircles in the alternating current impedance spectra. The semicircle at high frequency was assigned to the bulk resistance of the polymer electrolyte and its capacitance in parallel.^35^ The broad semicircle in the medium‐to‐low frequency region was attributed to interfacial resistance between lithium electrode and polymer electrolyte. The interfacial resistance was considered to comprise of a surface film resistance on Li and the charge‐transfer resistance of the Li^+^ + e^−^ = Li reaction. As can be seen from the inset of Figure 4b, the interfacial resistance changed from 1300 to 1370 Ω after polarization process. From Bruce–Vincent–Evans equation, we could easily obtain the lithium ion transference number of PVCA polymer electrolyte (0.57), much higher than PEO‐based polymer electrolyte (≈0.2).^12^, ^33^, ^36^ So, the high lithium ion transference number of PVCA polymer electrolyte is beneficial for an enhanced power capability.

A wide electrochemical stability window is a critical factor for high performance electrolyte of high voltage battery. Asymmetrical cells of Li/PVCA‐SPE/stainless steel were assembled to evaluate the electrochemical stability window of PVCA‐SPE. Figure 4c,d presented the linear sweep voltammetry of PVCA‐SPE at 25 and 50 °C, respectively. As shown in Figure 4d, the current density began to increase obviously at 4.5 V versus Li/Li^+^ at 50 °C. It corresponded to the start of oxidative decomposition of the electrolyte.^32^ So, PVCA‐SPE exhibited excellent electrochemical stability even at the elevated temperature. The excellent solid/solid interface compatibility would prevent further oxidation of electrolyte, which endow this solid electrolyte a better electrochemical stability, resulting in a better cycle performance of lithium batteries.

**Figure 5:**
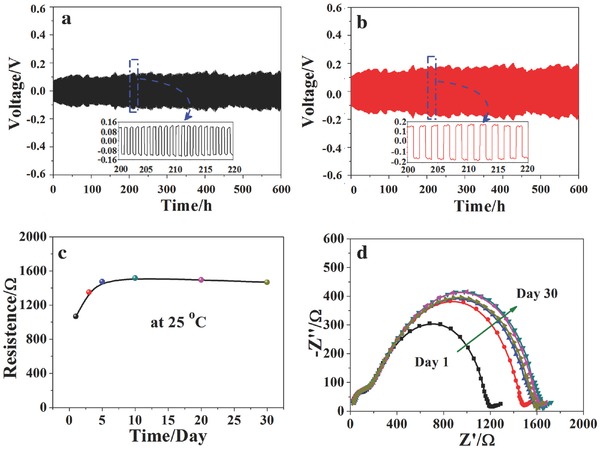
Chronopotentiometry results of Li/PVCA‐SPE/Li symmetrical cells at room temperature at the current density of a) 0.05 mA cm^−2^ for 0.5 h and b) 0.10 mA cm^−2^ for 1 h, respectively. Insets showed the magnified curves between 200 and 220 h; c) time dependence of the interfacial resistance of Li/PVCA‐SPE/Li symmetrical cell at room temperature; d) AC impedance spectra of Li/PVCA‐SPE/Li symmetrical cell.

The electrochemical compatibility of PVCA‐SPE/Li interphase was estimated by monitoring the impendence trend with a long‐term lithium deposition/striping cycles. **Figure** **5**a,b presented the cycling performance of symmetric Li/PVCA‐SPE/Li cells at a current density of 0.05 and 0.10 mA cm^−2^, respectively. The slight and synchronous voltage fluctuation could be observed in the deposition/striping process at the current density of 0.05 and 0.10 mA cm^−2^, which may be due to the fluctuation of room temperature. The over‐potential gradually increasing with time/or cycles may be attributed to the formation of a favorable SEI on the lithium metal surface. In addition, there was no observable short circuit phenomenon after 600 h polarization at both 0.05 and 0.10 mA cm^−2^. The sufficient rigidity of solid state PVCA‐SPE would suppress dendrite crossover during long‐term cycle and prevent short circuit occurrence, indicating a good compatibility between PVCA‐SPE and lithium anode.

The interfacial stability of PVCA‐SPE with lithium metal had also been evaluated by analyzing the impedance variation of the Li/PVCA‐SPE/Li symmetrical cell for a period of 30 d (shown in Figure 5c). The alternating current impedance spectra of symmetrical cell were shown in Figure 5d. There were two semicircles in the impedance spectrum, which was due to the bulk electrolyte resistance and interfacial resistance between lithium electrode and polymer electrolyte. As can be seen from Figure 5d, no obvious variation of bulk electrolyte resistance was found during the measuring process. The interfacial resistance between lithium electrode and polymer electrolyte would continually increase to some extent during the first 5 d, then kept quiet stable at about 1500 Ω after fifth day. The interfacial stability between PVCA‐SPE and lithium anode was favorable for excellent cycling performance. Moreover, ex‐site PVCA‐SPE and PEO‐SPE were obtained from traditional casting technique for comparison. Figure S7 in the Supporting Information displayed the result of ac impedance spectra of symmetrical cells. Ex‐site PVCA‐SPE showed the highest interfacial resistance among these three polymer electrolytes, due to its poor interfacial contact with lithium electrode. It was worth noting that the interfacial resistance of Li/PEO‐SPE/Li cell would decrease from 3400 to 2800 Ω when the cells were stored at 80 °C for 2 h. So, PVCA‐SPE obtained from in situ generation possessed improved interfacial compatibility with the electrodes.

### 2.3 LiCoO_2_/PVCA‐SPE/Li Battery Performances

**Figure 6:**
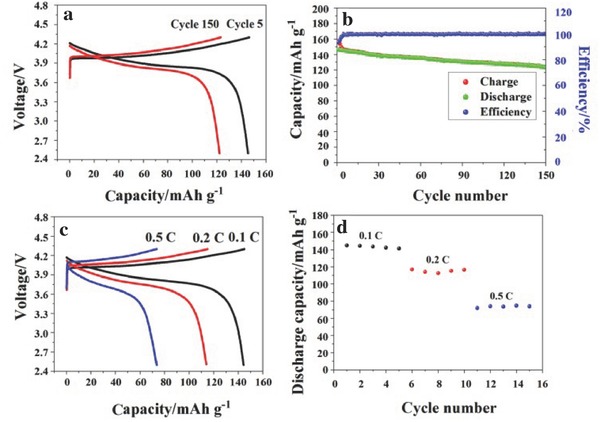
a) The charge/discharge curves of PVCA‐SPE based LiCoO_2_/Li cells at 5th and 150th cycles at 50 °C with the voltage range of 2.5–4.3 V; b) specific discharge capacity of PVCA‐SPE based LiCoO_2_/Li cells at a current density of 0.1 C with the voltage range of 2.5–4.3 V according to cycles; c) charge/discharge curves of LiCoO_2_/PVCA‐SPE/Li cells at varied current densities at 50 °C; d) discharge capacity of LiCoO_2_/Li cells using PVCA‐SPE at varied current densities.

The electrochemical performance of the PVCA‐SPE based lithium batteries was evaluated by using high voltage LiCoO_2_ (4.3 V) as the cathode and Li metal as the anode. The preparation of solid polymer lithium batteries via an in situ generation process was represented in Figure S8 in the Supporting Information. The batteries were stored at 60 °C for 24 h and 80 °C for another 10 h. Figure S9 in the Supporting Information presented the charge/discharge curves of PVCA‐SPE based LiCoO_2_/Li cells at a current density of 0.1 C (15 mA g^−1^) at 25 °C. The LiCoO_2_/Li battery delivered reversible capacity of about 97 mAh g^−1^ at a current density of 0.1 C, which was due to the low ionic conductivity of PVCA‐SPE and large polarization at 25 °C. However, PEO‐based LiCoO_2_/Li cells had a lower discharge capacity of 25 mAh g^−1^ (seen in Figure S9, Supporting Information). PEO‐based solid polymer electrolyte cannot satisfy the general conditions making lithium batteries operate at room temperature, although previous approaches have been investigated.^37^, ^38^ It was noteworthy that PVCA‐SPE based LiCoO_2_/Li battery exhibited a high discharge capacity and an excellent cycling performance at a constant current density of 0.1 C at 50 °C (shown in **Figure** **6**a,b). The initial discharge capacity was 146 mAh g^−1^ with an initial coulombic efficiency of 93.5%. After a fifth charge/discharge cycle, the coulombic efficiency would reach to a stable value about 99.3%. After the 150th cycle, the discharge capacities of PVCA‐SPE based LiCoO_2_/Li cell was 123 mAh g^−1^, corresponding to 84.2% of the initial capacity (146 mAh g^−1^), which was similar to the previous reports.^37^ The high capacity retention indicated good electrode/electrolyte interfaces stability and excellent cycling performance during the long‐term cycles. Figure S10 in the Supporting Information displayed the AC impedance spectra and its equivalent circuit of PVCA‐SPE based LiCoO_2_/Li battery at full‐charged state of first cycle, third cycle, and tenth cycle. AC impedance spectra of PVCA‐SPE based LiCoO_2_/Li battery showed that the resistance of LiCoO_2_/Li battery would slightly increase with the cycle number. The increased resistance mainly derived from the formation of passivation film between lithium metal and PVCA‐SPE interface. Fortunately, the resistance becomes stable after third cycle process, which was very important for long cycle performance of PVCA‐SPE based LiCoO_2_/Li battery.

As we know, the durable rate capability of polymer electrolyte in power battery is a critical performance. Figure 6c depicted the charge/discharge profiles of LiCoO_2_/PVCA‐SPE/Li batter**y** with the voltage range of 2.5–4.3 V, wherein the charge/discharge current densities varied from 0.1 to 0.5 C. The corresponding discharge capacity with five cycles performed at each current density of LiCoO_2_/Li batteries was shown in Figure 6d. A reversible discharge capacity of 114 mAh g^−1^ was achieved at 0.2 C, which was about 78% of the capacity at a rate of 0.1 C. Significantly, the LiCoO_2_/PVCA‐SPE/Li batter**y** could deliver a reversible discharge capacity of 73 mAh g^−1^ at 0.5 C, which was due to the excellent interface compatibility between polymer electrolyte and electrode. Based on the analysis above, it was believed that PVCA would be a promising solid polymer electrolyte for high energy lithium batteries.

**Figure 7:**
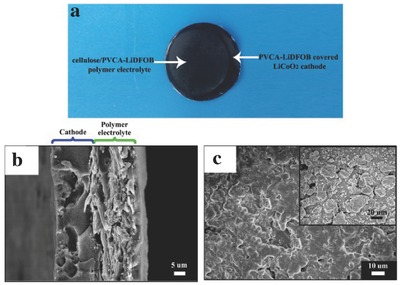
a) The digital image of LiCoO_2_ electrode and PVCA‐SPE after disassembly; b) the cross‐section SEM micrograph of LiCoO_2_ electrode with PVCA‐based polymer electrolyte (with cellulose); c) surface SEM micrograph of LiCoO_2_ electrode coated with PVCA‐LiDFOB polymer electrolyte (without cellulose). Inset was the surface SEM micrograph of pristine LiCoO_2_ electrode.

In order to find out the morphology changes of LiCoO_2_ electrode and PVCA‐SPE, the cell with a smaller cellulose nonwoven than LiCoO_2_ electrode (seen in Figure S11, Supporting Information) was disassembled after in situ polymerization of PVCA and its digital image was shown in **Figure** **7**a. SEM was used to investigate the microstructure of the cross‐section of LiCoO_2_ electrode with PVCA‐SPE and the surface morphology of PVCA‐LiDFOB coated LiCoO_2_ electrode. From Figure 7b, it could be seen that there was a compact adhesion between LiCoO_2_ electrode and polymer electrolyte. Some polymer electrolyte was even incorporated into the porous cathodes originated from the in situ polymerization of PVCA on the surface or even inside of the cathode (shown in Figure 7c). These findings manifested that close contact between the SPE and cathode interface would be greatly beneficial for impedance reduction and charge/discharge improvement.

**Figure 8:**
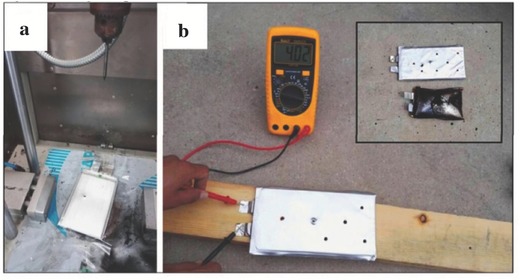
The consecutive nail penetration tests of the pouch type batteries using PVCA‐SPE as solid electrolyte and safety comparison between pouch type cells using PVCA‐SPE and conventional liquid electrolyte after nail tests.

Safety is a key concern for lithium batteries, especially for large energy storage equipments, such as electric vehicles and smart power grids. In order to evaluate the safety characteristics of PVCA‐SPE, the pouch type batteries using PVCA‐SPE as solid electrolyte were assembled. The preparation process of pouch type batteries was similar to that of traditional liquid electrolyte based pouch type batteries. After heating process at 60 °C for 24 h and 80 °C for 10 h, the pouch type battery was charged to 4.3 V at 50 °C. Then, the battery endured six consecutive nail penetration tests. It is worthwhile to note that the battery kept a good shape without any flame and explosion and displayed a relative high voltage at 4.02 V without short circuit after nail tests (seen in **Figure** **8**). By a sharp contrast, the pouch type battery using conventional liquid electrolyte released flame and underwent drastic expansion. These results were powerful evidences to prove that pouch type battery using in situ generation of PVAC‐SPE without flammable‐electrolyte present inherent safety.

## 3 Conclusion

In this work, a kind of novel PVCA‐based solid polymer electrolyte was successfully prepared by a facile in situ radical polymerization process, which possessed both interfacial compatibility toward lithium anode and high‐voltage LiCoO_2_ cathode (4.3 V vs Li/Li^+^). The PVCA‐SPE displayed a superior electrochemical stability window up to 4.5 V versus Li/Li^+^ and decent ionic conductivity of 9.82 × 10^−5^ S cm^−1^ at 50 °C. The high voltage PVCA‐based solid‐state LiCoO_2_/Li batteries represented superior charge/discharge performance, considerable rate capability and excellent cyclic performance. The high voltage of LiCoO_2_ would endow the lithium ion batteries have a higher energy density (260 Wh Kg^−1^) than the previous LiCoO_2_/graphite (4.2 V) batteries. Although the ionic conductivity of PVCA‐SPE was only about 2.23 × 10^−5^ S cm^−1^ at room temperature, adding plasticizers, inorganic ionic conductor or nanoparticles, copolymerization would be effective method to improve ionic conductivity of PVCA‐based solid polymer electrolyte. It was demonstrated that in situ generated PVCA based electrolyte could be a very promising solid polymer electrolyte candidate for high energy solid state lithium batteries.

## 4 Experimental Section

*Synthesis of Materials*: 1.43 g LiDFOB (Innochem(Beijing) Technology Co., Ltd., 99.9%) was dissolved in to 10 mL VC (Energy Chemica., 99.9%) to get a homogeneous and transparent solution (1.0 m LiDFOB in VC, ≈9.6% (w/w)), then the solution was added 10 mg AIBN (Sinopharm Chemical Reagent Co., Ltd.). The solution was injected into 2032 lithium battery, where cellulose separator was adopted as Supporting Information for polymer electrolyte which separated cathode and anode. Later, the lithium batteries were kept constantly at 60 °C for 24 h and 80 °C for 10 h in hot box to generate the completion of polymerization of VC. The ultimate corresponding loading amount of PVCA‐LiDFOB in PVCA‐SPE was 0.013 mg cm^−2^. The obtained PVCA was purified after three cycles of a redissolution–reprecipitation method using dimethylformamide and diethyl ether as a solvent and precipitant, respectively. The calculated polymerization conversion of VC into PVCA was more than 95%.

In order to show the picture of PVCA‐SPE directly, a piece of cellulose paper with VC liquid electrolyte was sandwiched in between two glass panes. Then, the cellulose paper with VC liquid electrolyte was stored at 60 °C for 24 h and 80 °C for 10 h. A translucent PVCA‐SPE was prepared (shown in Figure 1b). In order to get the microstructure of PVCA solid polymer electrolyte, symmetric batteries stainless steel/PVCA‐SPE/stainless steel were fabricated. After heat process, the batteries were disassembled to get the PVCA‐SPE.

*Characterization*: The morphology of the PVCA‐SPE was investigated by a field emission scanning electron microscopy (Hitachi S‐4800 at 5 kV). ^1^H and ^13^C spectra of PVCA were recorded on a Bruker AVANCE III 600 MHz with tetramethylsilane as internal reference. Thermogravimetric analysis of PVCA‐LiDFOB was carried out on TG 209F1 Iris (NETZSCH). Differential scanning calorimetry of PVCA‐LiDFOB was conducted on DSC 200F3 (NETZSCH) from –60 °C to 200 °C. X‐ray diffraction data was collected with a Bruker‐AXS Microdiffractometer (D8 Advance) using Cu Kα radiation (λ = 1.5406 Å). Infrared spectra measurements were conducted on a Fourier transform infrared spectrometer (Bruker VERTEX 70).

*Electrochemical Measurements*: The ionic conductivities of PVCA‐SPE were determined via an AC technique. The electrolyte was sandwiched between two stainless steel. Data were acquired using BioLogic VSP‐300 with an AC voltage amplitude of 10 mV over the frequency range from 0.1 Hz to 1 MHz. The ionic conductivity of polymer electrolyte was calculated by using following equation

(2) $$\sigma =\frac{l}{S\;R}$$

where *l* presents the thickness of polymer electrolyte, *S* is the contact area between electrode and electrolyte, and *R* corresponds to the bulk resistance of polymer electrode.

Cyclic voltammetry and linear sweep voltammetry of PVCA‐SPE were tested on a Li/PVCA‐SPE/stainless steel cells by impedance spectroscopy at a scanning rate of 1 mV s^−1^. The lithium ion transfer number of PVCA‐SPE was calculated from Bruce–Vincent–Evans equation

(3) $$t_{\mathrm{Li+}}=\frac{I_{\mathrm{ss}}}{I_{o}}\;\cdot \frac{\left(V-I_{o}\;R_{o}\right)}{\left(V-I_{\mathrm{ss}}\;R_{\mathrm{ss}}\right)}$$

where *V* is the applied polarization voltage, *I*_o_ and *R*_o_ are the initial current and the initial interfacial resistance before polarization, respectively, and *I*_ss_ and *R*_ss_ are the steady‐state current and the steady‐state interfacial resistance after polarization for 10 000 s, respectively.

For cell performance tests, the LiCoO_2_ cathode was prepared in a conventional casting method, by mixing 80 wt% LiCoO_2_, 10 wt% acetylene black, and 10 wt% PVCA. Finally, the specific density of active material on the electrodes was about 1.5 mg cm^−2^. The galvanostatic charge/discharge tests of coin‐type cells (CR2032) were conducted on LAND testing system (Wuhan LAND electronics Co., Ltd.) at 50 °C.

## Supporting information

As a service to our authors and readers, this journal provides supporting information supplied by the authors. Such materials are peer reviewed and may be re‐organized for online delivery, but are not copy‐edited or typeset. Technical support issues arising from supporting information (other than missing files) should be addressed to the authors.

Supplementary
