# Supplementary material for: In Situ Generation of Poly (Vinylene Carbonate) Based Solid Electrolyte with Interfacial Stability for LiCoO2 Lithium Batteries
Source: Adv Sci (Weinh). 2016 Nov 10;4(2):1600377. doi: 10.1002/advs.201600377 (PMC5323859; doi:10.1002/advs.201600377)
Supplement: Supplementary file 1 — Supplementary [file ADVS-4-na-s001.pdf]

Copyright WILEY-VCH Verlag GmbH & Co. KGaA, 69469 Weinheim, Germany, 2016.

ADVANCED SCIENCE
Open Access

Supporting Information

for *Adv. Sci.,* DOI: 10.1002/advs.201600377

In Situ Generation of Poly (Vinylene Carbonate) Based Solid Electrolyte with Interfacial Stability for $LiCoO_2$ Lithium Batteries

Jingchao Chai, Zhihong Liu, Jun Ma, Jia Wang, Xiaochen Liu, Haisheng Liu, Jianjun Zhang, Guanglei Cui,* and Liquan Chen*

Copyright WILEY-VCH Verlag GmbH & Co. KGaA, 69469 Weinheim, Germany, 2016.

Supporting Information

In-situ Generation of Poly (vinylene carbonate) Based Solid Electrolyte with Interfacial Stability for $LiCoO_2$ Lithium Batteries

Jingchao Chai,[a,b] Zhihong Liu, [a] * Jun Ma,[a] Jia Wang,[a,b] Xiaochen Liu,[a, c] Haisheng Liu,[a] Jianjun Zhang,[a,b] Guanglei Cui[a] * and Liquan Chen[d]

a. Qingdao Industrial Energy Storage Technology Institute, Qingdao Institute of Bioenergy and Bioprocess Technology, Chinese Academy of Sciences, Qingdao 266101, China

b. University of Chinese Academy of Sciences, Beijing 100049, China

c. College of Chemistry and Molecular Engineering, Qingdao University of Science & Technology, 266042, Qingdao, China.

d. Beijing National Laboratory for Condensed Matter Physics. Institute of Physics, Chinese Academy of Sciences. Beijing 100190, China

** Corresponding author. Tel: 86-532-80662746, Fax: 86-532-80662744.*

E-mail: liuzh@qibebt.ac.cn, cuigl@qibebt.ac.cn

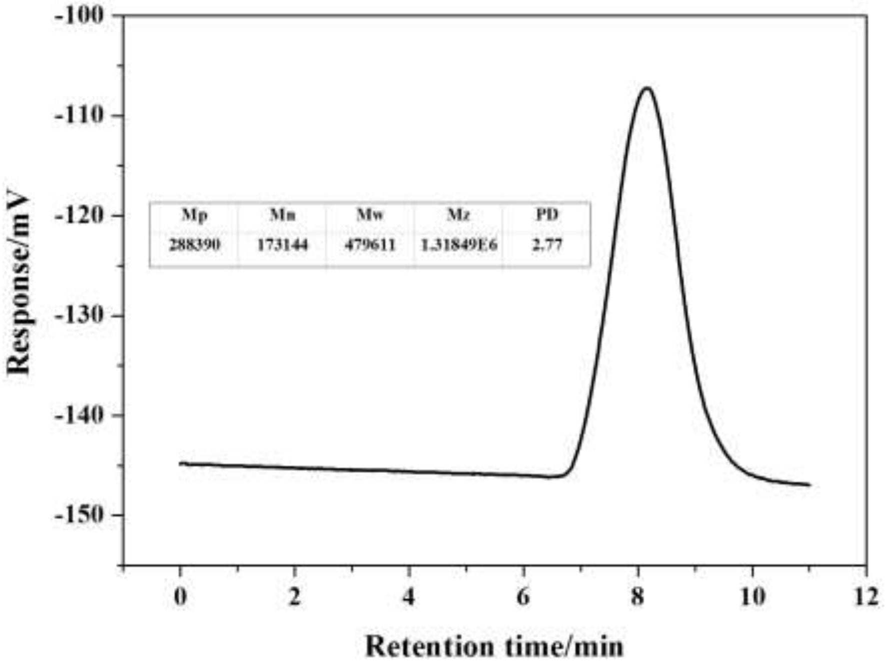

Figure S1. GPC result of PVCA.

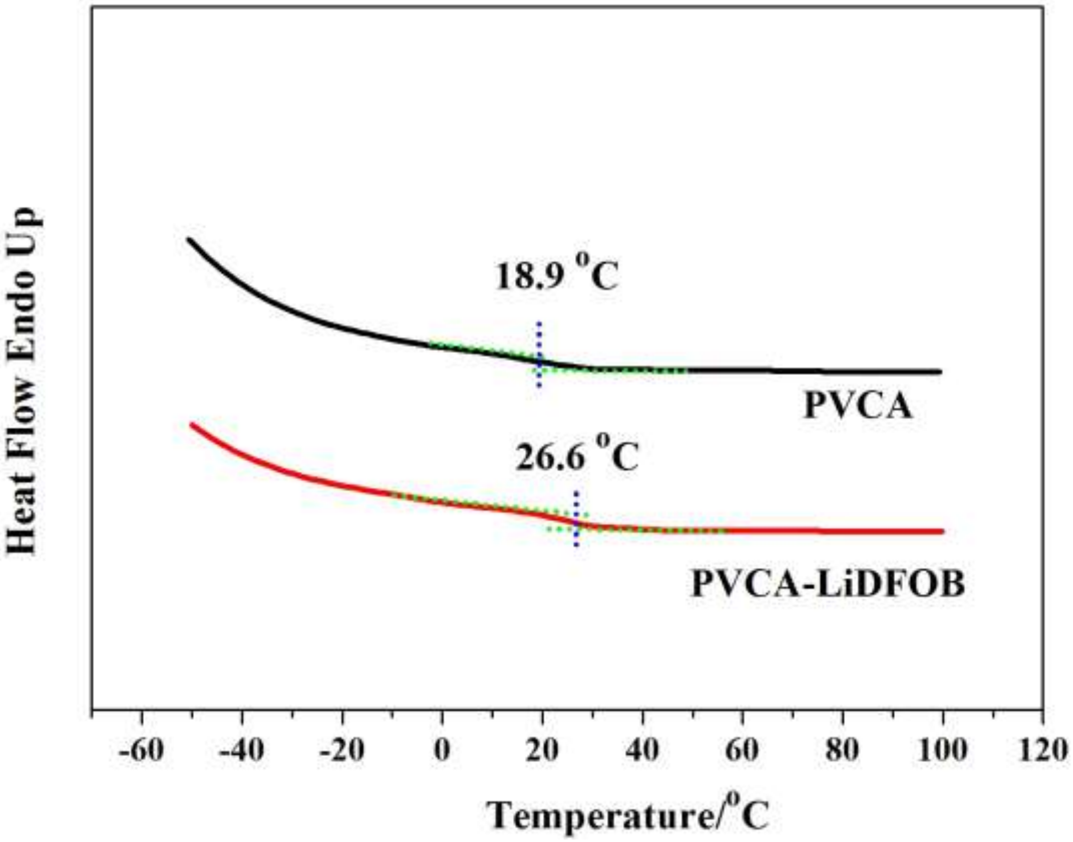

Figure S2. DSC curves of PVCA-SPE from -50 ^{o}C to 100 ^{o}C.

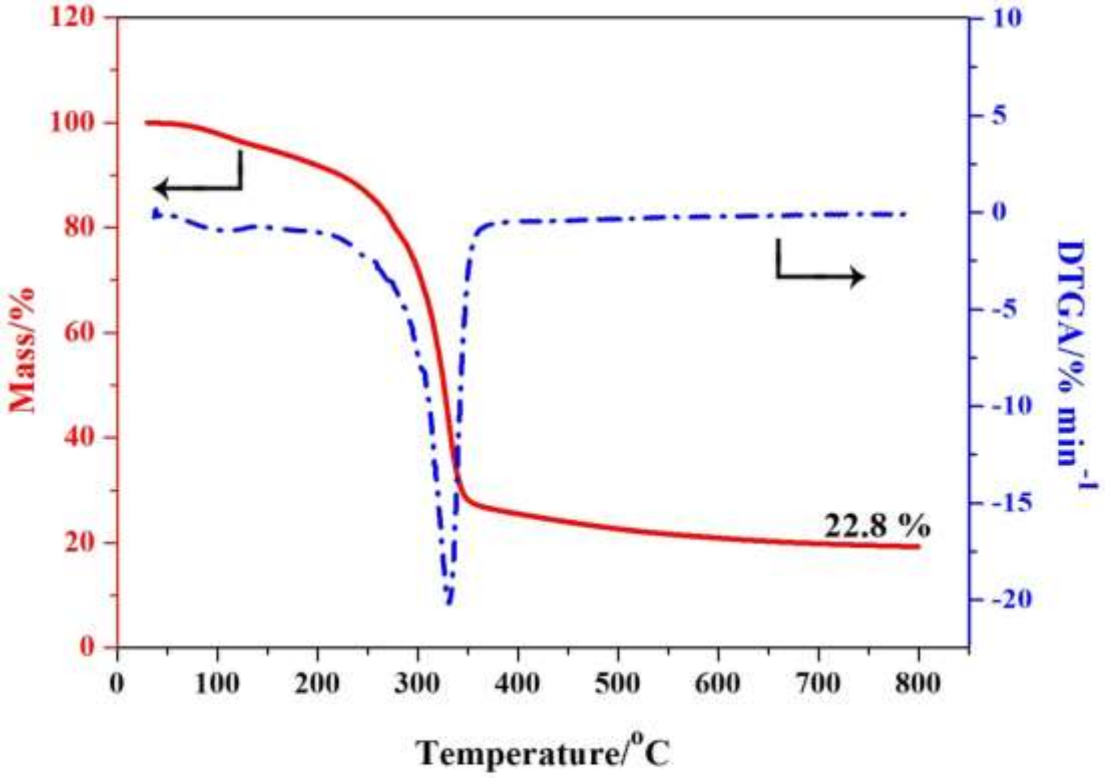

Figure S3. TGA curve of PVCA-SPE from room temperature to 800 °C.

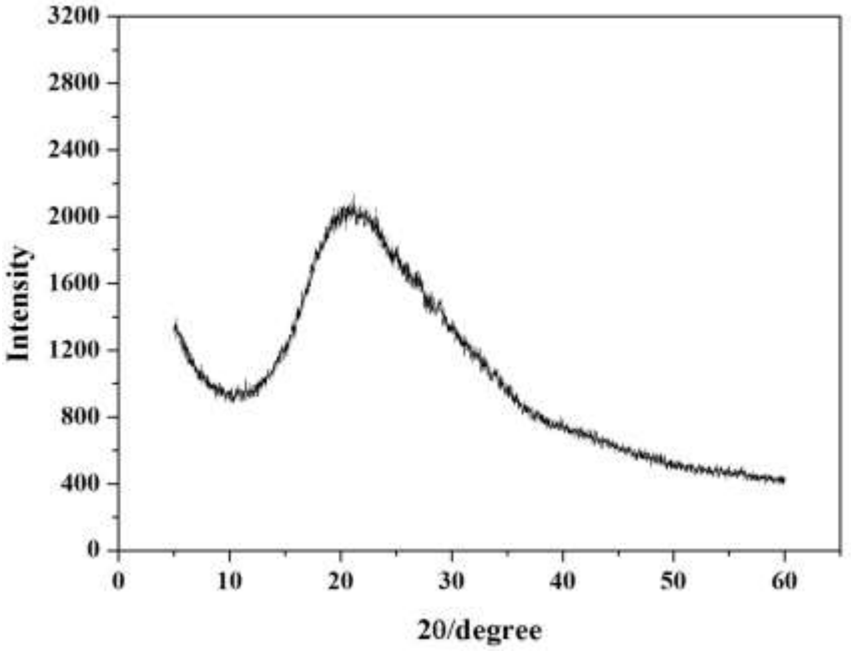

Figure S4. XRD curves of PVCA-SPE.

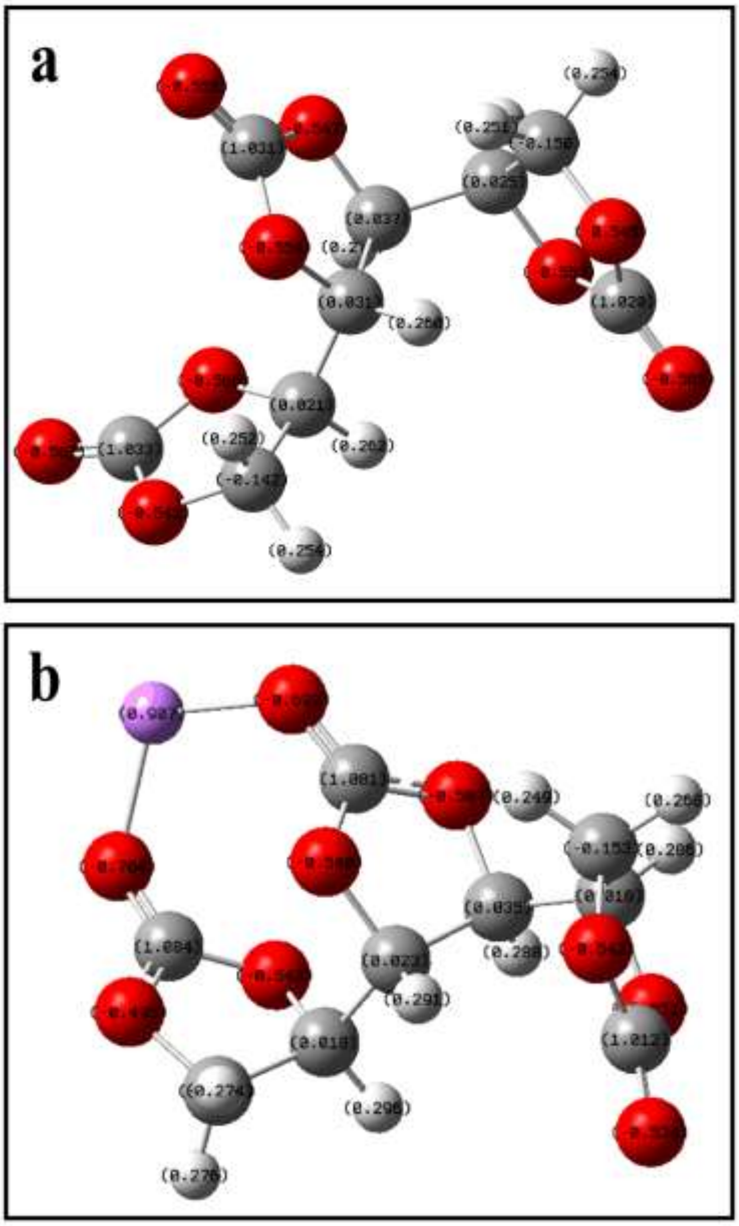

Figure S5. Natural bond orbital charge of PVCA without (a) and with (b) lithium ion.

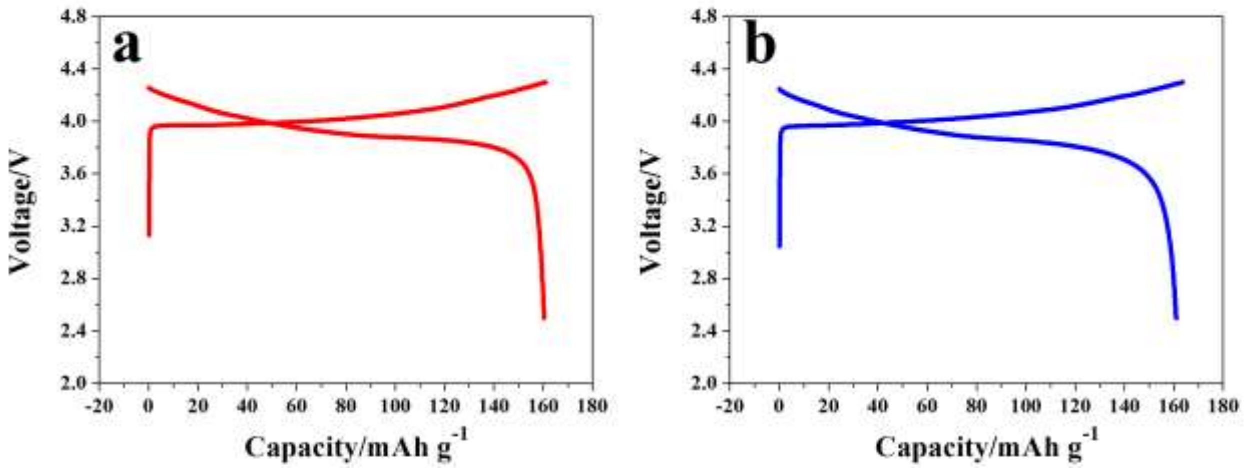

Figure S6. The charge/discharge curves of liquid electrolyte based $LiCoO_2$/Li batteries a) with and b) without AIBN (1.0 mg ml^{-1}).

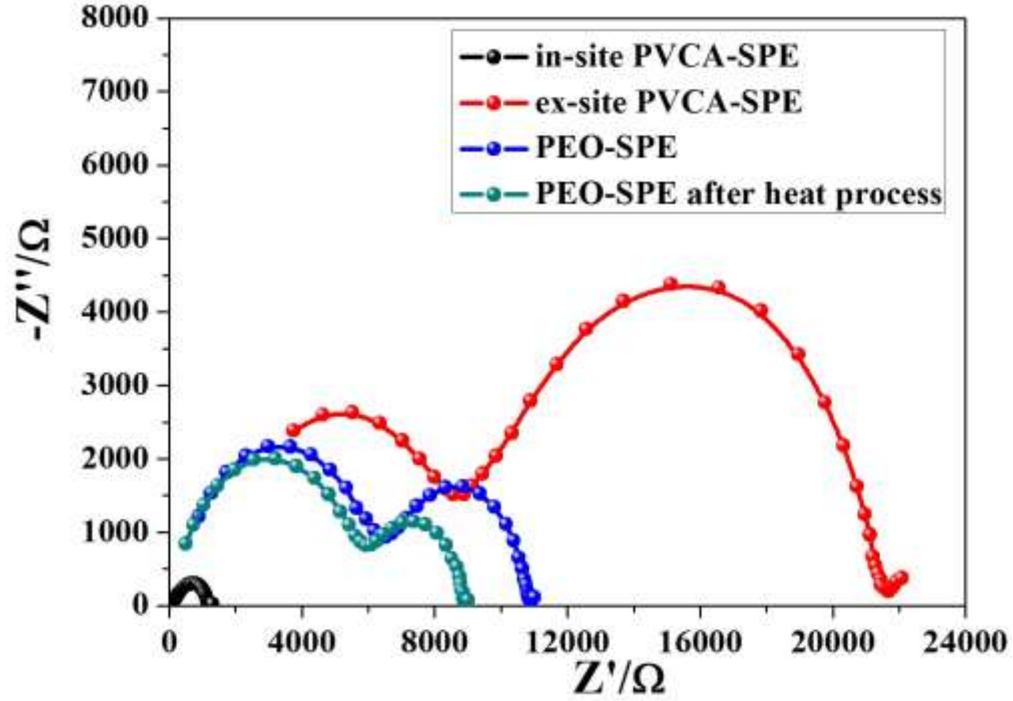

Figure S7. Ac impedance spectra of symmetrical cells using varied electrolyte.

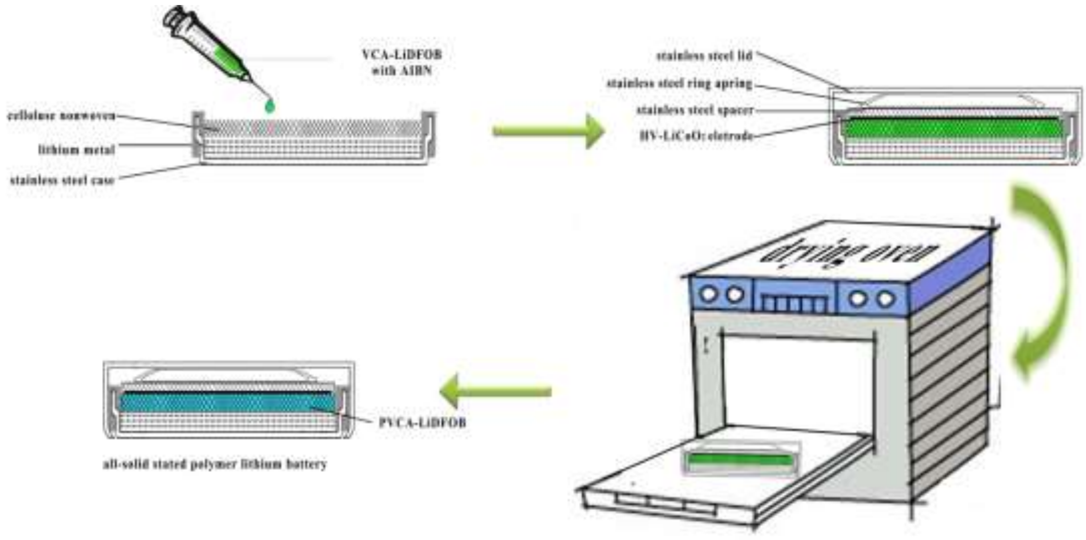

Figure S8. The preparation of solid polymer batteries by in site polymerization.

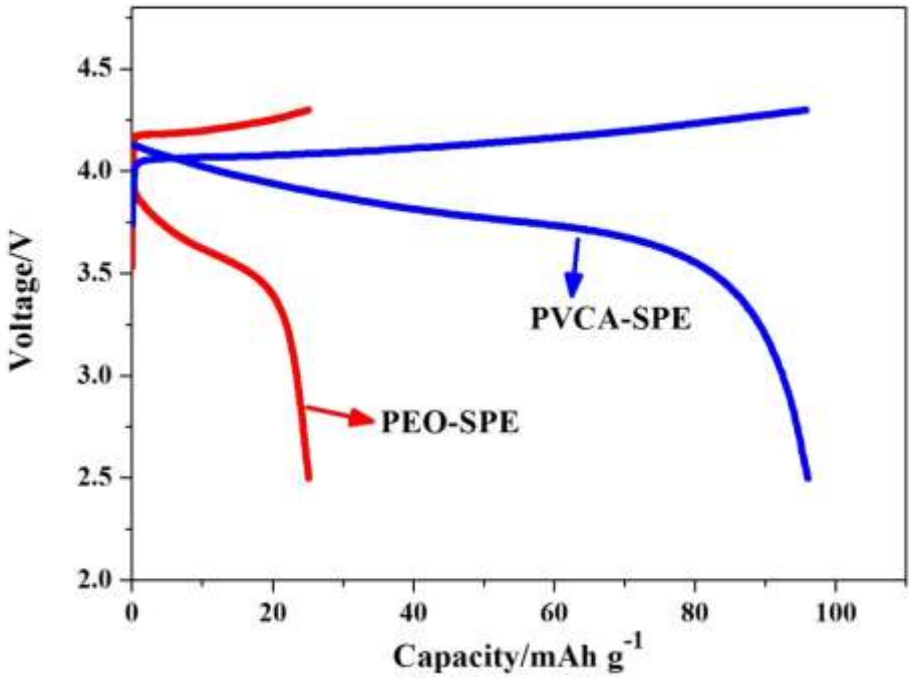

Figure S9. Charge/discharge curves of PVCA-based and PEO-based $LiCoO_2$/Li cells at 25 ^oC.

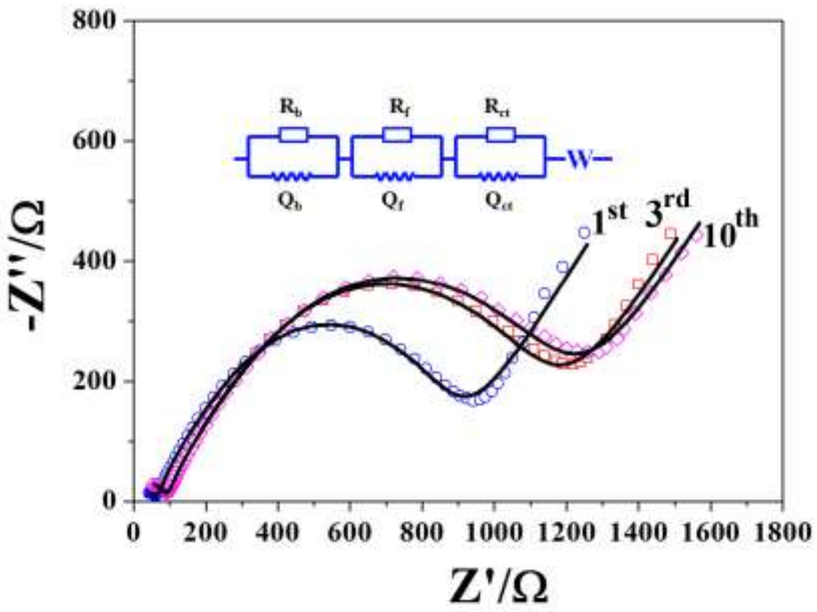

Figure S10. AC impedance spectra and its equivalent circuit of PVCA-SPE based $LiCoO_2$/Li battery at full-charged state of first cycle, third cycle and tenth cycle.

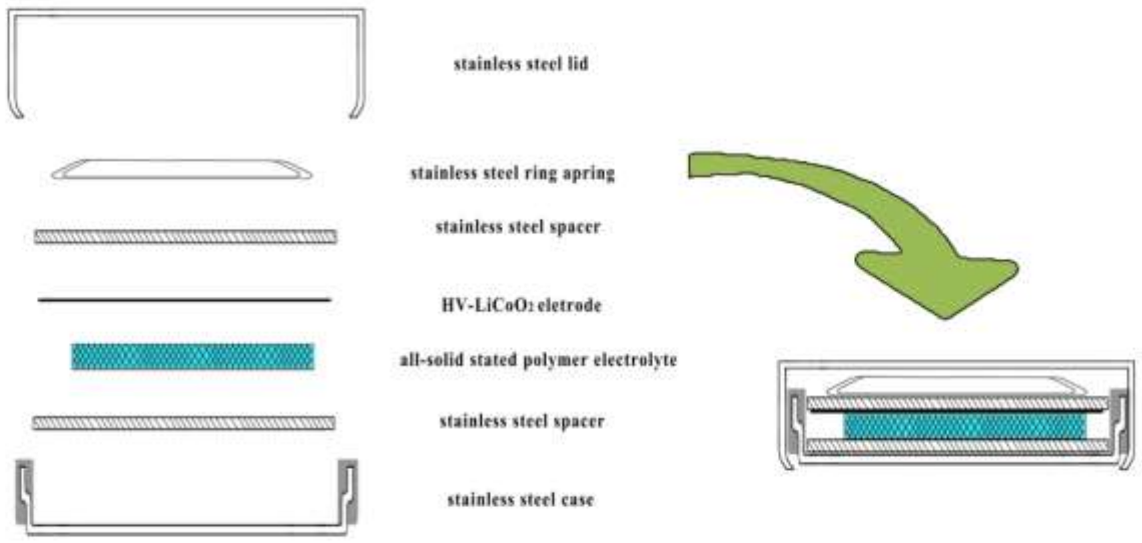

Figure S11. Preparation process of composited $LiCoO_2$ electrode with polymer electrolyte.
